# Should co-payments for financially deprived patients be lowered? Primary care physicians’ perspectives using a mixed-methods approach in a survey study in Tokyo

**DOI:** 10.1186/s12939-017-0534-x

**Published:** 2017-02-22

**Authors:** Machiko Inoue, Yuko Kachi

**Affiliations:** 1 Beth Israel Deaconess Medical Center, Harvard Medical School, 1309 Beacon St, Brookline, MA 02446 USA; 2 Department of Hygiene and Public Health, Nippon Medical School, 1-1-5, Sendagi, Bunkyo-ku, Tokyo 113-8602 Japan

**Keywords:** Co-payment, Financial burdens, Primary care physicians, Health-care equity, Japan

## Abstract

**Background:**

Japan’s stagnant economy has produced increasing income disparities, and the effect of poverty on health and health-care utilization is a significant issue. Co-payments could be a trade-off for patients when seeking medical care and limit health-care utilization. We sought primary care physicians’ experiences in dealing with financially deprived patients and their perspectives about reducing co-payments by low-income patients.

**Methods:**

We used a convergent mixed-methods approach in a mail survey; it was distributed to 1989 primary care physicians practicing in areas with the highest proportions of socially disadvantaged individuals in Tokyo. The survey items included an open-ended question, seeking the participants’ perspectives about reducing co-payments by low-income patients from the current 30%, and closed questions, asking their experience of patient behavior related to financial burdens during the previous 6 months.

**Results:**

We analyzed the responses of 365 physicians. Sixty-two percent of the primary care physicians agreed with lowering co-payments for financially deprived patients; however, the remainder disagreed or were uncertain. Those who disagreed were less likely to have experienced patient behavior related to financial burdens. The participants suggested challenges and potential measures for reducing co-payments by low-income patients in light of tight governmental financial resources and rapidly increasing health-care expenditures in Japan. The physicians were also concerned about the moral hazard in health-care utilization among patients receiving social welfare who obtain care at no cost.

**Conclusions:**

From their experience in having dealt with low-income patients, the majority of physicians were positive about lowering co-payments by such patients; the remainder were negative or uncertain. It may be necessary to raise awareness of patients’ socioeconomic status among primary care physicians as a possible deterrent for seeking care. To maintain health-care equity, policy makers should consider balancing co-payments among individuals with differing financial levels and health-care needs.

## Background

Japan has been internationally renowned for its universal health coverage, which has long enabled equitable access to health care [1, 2]. However, owing to the country’s longstanding stagnant economy [3], income disparities are increasing in Japan; accordingly, the impact of poverty on health and health-care utilization has become a highlighted issue [4]. Japan’s relative poverty rate has continued to rise since 1985, and its highest-ever figure of 16.1% was recorded in 2012 [5]. In that year, one in six Japanese lived in relative poverty, with incomes of under half of the median equivalent disposable income, i.e., 1.22 million yen (about US$11,700) [6]. Since its launch in 1961, universal health coverage has undergone multiple reforms to meet rapidly changing social needs. Under Japan’s current policy, all patients aged under 70 years have to pay 30% of the total health-care costs—regardless of their income—as a co-payment every time they see a primary care doctor [1]. The same co-payment applies to outpatient and inpatient care, prescriptions, diagnostic tests, and surgery. Since co-payment is not a fixed amount, patients with complex, multiple long-term conditions face a greater financial burden when receiving medical care. A reimbursement system (High-Cost Medical Expense Benefit) exists for medical payments that exceed a set monthly threshold; that system was revised in 2015 to be in line with a patient’s household income [7]. For example, patients do not have to pay more than 57,600 yen (about $550) a month out of their own pockets if their household income is below 3.7 million yen (about $35,500) [7]. The exception is for individuals receiving public social welfare: they can obtain medical care at no charge, all such care being covered by the government. In Japan, people receiving social welfare comprised 1.7% of the total population in 2014 [8]. Most individuals in relative or absolute poverty did not receive social welfare: some failed to meet the criteria for receipt; however, many others did not choose to receive it owing to the social stigma or sense of shame.

A current issue is the non-payment of health insurance premiums through financial burdens on deprived households [2]. Specifically, among individuals eligible for the government-run health insurance program (covering almost 40% of the total population; most of those eligible are self-employed or unemployed), 16.7% do not pay the premium regularly [9]; that could result in losing eligibility for insurance. In addition to people at risk of being uninsured, insured individuals with financial insecurity may refrain from receiving medical care owing to the required co-payment at each health-care visit.

Co-payments may operate as a trade-off for patients when seeking medical care, and they affect the amount of health-care utilization [10]. In countries with universal health coverage, such as France, patient cost sharing has been reported to reduce the frequency of physician visits; that was especially true for patients in low social classes [11]. As a result of rapid increases in patient charges when consulting a general practitioner before 1995, more than half of financially deprived patients in Sweden reported that they had forgone medical care owing to costs at least once during the previous year; those who had forgone care perceived worse personal health conditions [12]. In Japan, reports differ as to how often people forgo medical care; however, it is known that low-income individuals more likely forgo or delay seeking care owing to costs compared with the high- or middle-income population [13–15]. The socially deprived population, though, has poorer health [16] and more unmet care needs [17–19]. To achieve optimal outcomes, patients with chronic illnesses, such as hypertension and diabetes, particularly need to make regular health visits and adhere to treatment. Cost-related medication non-adherence is strongly associated with worse health outcomes, such as hospitalization and death in patients with cardiovascular disease [20, 21].

In Japan, physicians are not legally allowed to make repeat prescriptions without seeing the patient [22]; thus, patients must see their doctor every time they need a prescription. For financially deprived patients, the cost and benefit of seeking care is a trade-off; accordingly, if patients are asymptomatic, non-adherence to treatment can easily occur [23–25]. That being the case, primary care physicians may be the first to notice that a patient’s financial burdens are hindering them from receiving necessary medical care. Those physicians should be responsible for arranging a more acceptable treatment plan with patients to prevent loss to follow-up and non-adherence [26–28]. Thus, primary care physicians’ perspectives and attitudes regarding the care of low-income patients play an important role in mitigating the inequality of health-care utilization related to economic status.

To the best of our knowledge, no studies have addressed this topic in Japan. Accordingly, to examine experiences and perspectives in seeing patients with financial issues, we conducted a survey of primary care physicians practicing in especially deprived areas in Tokyo; we obtained both quantitative and qualitative data. We have previously published a paper using part of the results of that survey. We reported that approximately 90% of the surveyed physicians had experience of seeing patients with financial burdens; they had made efforts to meet patient expectations by somehow lessening those burdens with respect to the medical care they provided [29]. In the present paper, we use the remaining survey results to report primary care physicians’ perspectives about lowering the co-payment amount for low-income patients from the current 30%. Employing a convergent mixed-methods approach, we compared the themes derived from the qualitative responses using quantitative data from the respondents to expand our understanding of physician characteristics and perspectives.

## Methods

### Design, setting, and participants

We conducted a mail survey among primary care physicians practicing in especially deprived areas of Tokyo from July to September 2014. We used a convergent mixed-methods approach, whereby we collected qualitative and quantitative data simultaneously and then integrated them for interpretation [30]. We selected the survey areas as the 12 municipalities with the highest proportions of residents on social welfare among the 69 in Tokyo. The participants were physicians working in clinics in those areas that included the specialty of internal medicine; we requested that one physician of the highest rank respond on behalf of each clinic. We distributed 1989 questionnaires.

### Ethical considerations

Before answering the questionnaire, the respondents read the explanation of the survey and understood that participation was voluntary. We considered return of the completed questionnaire anonymously to signify consent to participate. After returning the questionnaire, each respondent received a gift card equivalent to 1000 yen (about $9.5). The study protocol was approved by the institutional review board of Nippon Medical School (No. 25-16), Tokyo, Japan.

### Survey items

We included an open-ended question, asking the participants’ perspectives about lowering the co-payment amount for low-income patients from the current 30% in the context of hypothetical legislature. We also included the following closed-ended questions to assess physicians’ experience and background. First, we asked if during the previous 6 months, they had experience of patient behavior related to financial burdens, such as loss to follow-up, medication non-adherence, postponing necessary tests, or refusing specialist referrals. Second, we assessed whether the physicians explained medication costs to patients in terms of “always,” “usually,” “sometimes,” “seldom,” or “never.” Third, we evaluated the self-perceived decision-making style using three categories: paternalism, informed consent, and shared decision making. Paternalism signified the physician deciding without asking the patient’s opinion or the patient consenting to the decision made by the physician without being presented with other options. Informed consent referred to the patient consenting to what the physician thought best after being presented with alternative options. Shared decision making signified the presentation of possible treatment options and the physician discussing the matter with the patient before the decision being made. We also collected physician demographics, such as age, sex, and specialty.

### Analysis and integration of qualitative and quantitative results

We first compared the demographics of the respondents in the open-ended question with the overall survey respondents. To derive underlying themes, we then thematically analyzed the text responses to the open-ended question (qualitative data), which led to the development of several categories. We held discussions to agree upon the development of themes and categories. Based on the developed categories, we chose two groups of respondents: those who responded “agree” or “necessary” and those who responded “disagree” or “unnecessary” to the qualitative question about lowering the co-payment amount for low-income patients. We compared the characteristics of those two groups according to the quantitative data using chi-square tests, i.e., demographics, experience of seeing patients with financial burdens, explaining medication costs to patients, and decision-making styles. We performed statistical analyses using IBM SPSS Statistics version 23 (IBM Corp. New York, USA). We set statistical significance at *P* <0.05. Finally, as the integration phase in the mixed-methods approach [31], we expanded our understanding of the patterns in the physician characteristics and their perspectives based on our interpretation of both the qualitative and quantitative data.

## Results

Among the 617 questionnaires returned (response rate, 31.0%), 550 were complete and used for the overall analyses. Among those, 365 participants responded to the open-ended question, and we used their data for qualitative analyses. Table 1 shows the characteristics of the two groups; no remarkable differences are evident between them. Over half of the participants had experience of patient behavior related to financial burdens during the previous 6 months, such as loss to follow-up and requests to postpone necessary tests. In terms of decision-making styles, the majority of respondents employed paternalism or informed consent; shared decision making was uncommon. More than half of respondents explained medication costs to patients sometimes or most of the time.

**Table 1:** Characteristics of the respondents to the qualitative question and total participants

	Respondents to the qualitative question (*N* = 365)	Total participants (*N* = 550)
Gender – male, %	83.6	82.6
Age groups (years), %		
30–39	3.3	2.9
40–49	19.7	19.1
50–59	38.9	38.7
60 and over	38.1	39.3
Specialty – general practice, %	51.2	49.5
Experience of patients’ behaviors related to financial burdens during the past 6 months – any, %		
Loss of follow-up	54.8	50.2
Medication nonadherence	48.2	43.5
Postponing necessary tests	58.6	53.8
Refusing referral to specialist	22.0	18.6
Decision making style, %		
Paternalism	40.5	45.3
Informed consent	42.2	40.0
Shared decision making	15.1	14.7
Explaining the cost of medication to patients – always, usually, or sometimes, %*	55.6	54.2

*Always, usually, or sometimes as opposed to seldom or never

### Qualitative results

Table 2 presents the primary care physicians’ perspectives about lowering out-of-pocket payments for low-income patients. They are grouped into four categories: agree/necessary; disagree/unnecessary; possible measures and challenges for implementing the legislation; and need to change the current redemption system of providing free care for those on social welfare.

**Table 2:** List of themes of physicians’ perceptions about possible legislation for lowering out-of-pocket payments by low-income patients

Category	Themes	Quotes
Agree/necessary	Equal access to health care	“High out-of-pocket payments prevent patients from seeking necessary medical care, which leads to poorer health.” “If they are asymptomatic, low-income patients soon stop taking medications. Those patients definitely need health education.”
	Better health necessary for better work status	“Receiving appropriate health care will enable patients to get a better job, which will lead to a better life.”
	Reducing future health-care costs	“Poorer health will lead to more ER visits and hospitalization, which will eventually result in increased overall health-care costs.”
Disagree/not necessary	Sharing equal burden in receiving care	“To avoid inequality and misuse of health-care benefits, the rate charged should be the same for everyone.”
	Responsibility in taking care of one’s own health condition and improving lifestyle	“If patients pay less for medical care, they will be less motivated to improve their lifestyles.” “Patients should spend more on medical care and quit spending on cigarettes and alcohol.”
	No need or merit	“The existing redemption systems are sufficient to protect the poor. Utilizing those systems should be enough.” “Other measures could be used to mitigate the burden for low-income patients.”
Challenges and potential measures for implementing the legislation	Tight government resources for health-care expenditure	“Any additional increase in health-care expenditure will make the government unable to maintain universal health coverage.” “Some measures need to be undertaken about the issues of high, rising drug costs.”
	Limiting the redemption for specific diseases and expensive treatment	“Expensive treatment, including diabetes medication such as insulin and some anti-cancer drugs, should be affordable to all patients who need it.”
	Potential risk of overutilization	“Free or low out-of-pocket payments might cause overutilization of medication. That is seen in patients on social welfare, who can get medicines for free, and should be avoided.”
Necessity to change the current policy of patients on social welfare paying no charges	Wide gaps in the payment burden between those on social welfare and those not	“Even if their income is at the same level, people on social welfare can receive care for free, but others are charged the 30% co-payment. This gap is too big. Patients on social welfare should pay something for receiving care.”
	Moral hazard in health-care utilization among patients on social welfare	“Free care is problematic. Patients take it for granted.” “Patients come to see doctors too often and demand unnecessary tests and medications.”

First, 62.2% of participants (227/365) responded that they agreed it was necessary to lower out-of-pocket payments for low-income patients not on social welfare. The participants mentioned their experience as physicians of losing patients to follow-up, medication non-adherence, and refusing necessary tests, referrals, or treatments. The respondents recognized the relationship between financial burdens and patient behavior as well as the importance of ensuring equal access to health care. The participants stated that they were trying to mitigate the burden on patients by making the consultation interval longer and choosing cheaper drugs. They stated that they felt frustration when negotiating with patients to accept proper care. Further, the physicians recognized that better health could help patients obtain a better-paid job, thereby alleviating financial burdens, and that receiving medical care and health education were necessary in that regard. Moreover, the participants considered that ensuring necessary medical care for low-income patients would prevent worsening of their conditions, resulting in reduced future health-care costs.

Second, one group of physicians (76/365) did not agree with the necessity of lowering out-of-pocket payments. Those doctors considered that patients should share an equal burden in receiving medical care and that lowering the payments for low-income patients would cause inequality with that burden. They also believed that patients should assume responsibility in taking care of their physical condition and improve their lifestyles, such as with smoking and alcohol consumption, which are associated with low incomes. Those physicians regarded payment for medical care as a kind of brake and that it motivated patients to improve their lifestyles. Some physicians thought there was no need or merit in lowering co-payments: they noted that various measures were already in place to mitigate financial burdens for patients with intractable diseases and that utilizing existing measures was sufficient; introducing new measures was unnecessary.

Third, the remaining participants (62/365) were uncertain about the feasibility of legislation to lower out-of-pocket payments for low-income patients; they believed that its implementation would present a number of challenges. Most of all, these physicians were concerned that any measures to mitigate the burden for low-income patients would be impractical owing to the Japanese government’s tight financial resources; the physicians believed that such measures would create a dilemma when treating such financially stressed patients. These respondents considered that when discussing affordable treatment with low-income patients so as to avoid loss to follow-up or medication non-adherence, their comments would express disappointment or a sense of hopelessness with the government’s financial constraints. The physicians in this group suggested potential measures, such as limiting the redemption for specific diseases and expensive forms of treatment, rather than applying redemption for all patient care costs regardless of the treatment burden. For example, costly medications for diabetes, including insulin, would be particularly problematic in the case of poor adherence. The potential risk of overutilization of health care was another concern raised: some physicians believed that providing free care should be avoided by all means; they mentioned the current issue of health-care overutilization by patients on social welfare who receive care for free.

Fourth, some physicians (50/365) expressed the opinion that some action should be taken to address the current policy of patients on social welfare having to pay no costs. There is a large discrepancy in the out-of-pocket expenses for visiting a doctor among financially deprived patients: low-income patients have to pay 30% of the total cost, whereas patients receiving social welfare do not have to pay at all. Patients and physicians find this gap in the payment burden to be unfair. On a daily basis, physicians observe the moral hazard in health-care utilization by people receiving social welfare: they stated that such patients take the free care for granted. They also pointed out the existing moral hazard for physicians, noting that some physicians tend to order unnecessary tests or overutilize interventions; that is partly because patients demand them and partly because physicians can simply earn more based on the fee-for-service reimbursement system for outpatient care.

### Quantitative results

Table 3 shows the quantitative results, comparing the two groups who responded “agree” or “necessary” and “disagree” or “unnecessary” to the qualitative question we asked. There were no significant differences between the two groups in terms of age, specialty, or decision-making style. However, physicians who disagreed with lowering out-of-pocket expenses for low-income patients or considered it unnecessary were less likely to have had experience of patients’ loss to follow-up during the previous 6 months (42.1% vs. 58.0%, *P* = 0.016), which is one of the common behaviors of patients related to financial burdens. Moreover, those physicians were less likely to explain the cost of medication to patients always, usually, or sometimes (43.4% vs. 58.1%, *P* = 0.026).

**Table 3:** Comparison of characteristics of respondents between those who agreed and disagreed with the idea of lowering out-of-pocket payments by low-income patients

	Agree/necessary (*N* = 227)	Disagree/not necessary (*N* = 76)	*P* value*
Gender – male, %	85.5	76.3	0.065
Age groups (years), %			0.173
30–39	1.8	5.3	
40–49	22.0	17.1	
50–59	41.0	34.2	
60 and over	35.2	43.4	
Specialty – general practice, %	52.9	52.6	0.972
Experience of patients’ behaviors related to financial burdens during the past 6 months – any, %			
Loss of follow-up	58.0	42.1	0.016
Medication nonadherence	51.6	40.8	0.105
Postponing necessary tests	62.4	51.3	0.089
Refusing referral to specialist	26.5	15.8	0.057
Decision making style, %			0.826
Paternalism	39.4	41.3	
Informed consent	44.3	45.3	
Shared decision making	16.3	13.3	
Explaining the cost of medication to patients – always, usually, or sometimes, %**	58.1	43.4	0.026

*Chi-square test

**Always, usually, or sometimes as opposed to seldom or never

## Discussion

This study examined physicians’ experiences and perspectives regarding patients with financial issues. To the best of our knowledge, it is the first investigation to explore Japanese physicians’ perspectives about reducing co-payments for low-income patients from the current 30%. The majority of respondents agreed with the necessity of such a reduction, though a minority considered it unnecessary. Participants regarded the feasibility of implementing this reduction an area of huge concern. The physicians who agreed with the reduction or believed it necessary were more likely to have had experience of patients with financial burdens than those who disagreed with it or believed it unnecessary. Over the half of the physicians had experienced patient behavior related to financial burdens, such as loss to follow-up and medication non-adherence during the previous 6 months. These results indicate that the physicians believed the Japanese health-care system to be inequitable for the poor. Most of the primary care physicians who realized that patients’ economic status could deter them from undergoing necessary medical care discussed the situation with the patients so as to keep the out-of-pocket payments as low as possible toward avoiding loss to follow-up or non-adherence. Though the physicians aimed to provide the best possible care, they also felt frustrated and powerless because the patients’ economic status could not improve.

It was remarkable that some physicians clearly considered lowering the co-payments for financially deprived patients to be unnecessary. Combining our qualitative and quantitative data, we could infer that such physicians were less likely to explain the cost of medication to patients and may have missed signs of the patients’ wish to have the medical care-related costs reduced as much as possible. The idea that it is an individual’s responsibility to maintain good health through appropriate behavior and to take care of their own physical condition seemed to be prevalent among the participants; physicians who thought this way believed that all patients should accept the same financial burden for medical care regardless of their physical or financial conditions. This notion is related to “victim blaming” [32] and is incongruent with current understandings about the social determinants of health [33, 34]. One possible reason for this result is the diverse backgrounds of primary care physicians in Japan. Nearly 80% of respondents in this survey were aged 50 years or more, and may not have been exposed to Engel’s biopsychosocial model [35, 36]. Most of the primary care physicians in Japan had previously been trained as subspecialists but became primary care physicians in their later careers, which has been observed and called a “Two Career model of specialization” by Saigal and colleagues [37]. Therefore, it is likely that even the younger physicians have received little formal training that includes the skills to build a longitudinal trusting relationship with patients using a biopsychosocial model for understanding patients, the training officially started in the mid-2000s by the Japanese Academy of Family Medicine; that was later integrated to form a new academic body, the Japan Primary Care Association, in 2010 [38]. Primary care physicians clearly have an important role in treating socially deprived individuals and providing appropriate care from both medical and psychosocial aspects. Promoting the importance of social determinants of health should be emphasized in continuous medical education for primary care physicians [39].

Co-payments affect health-care utilization, especially among low-income individuals [10, 12, 40]. Our results indicate that the participants considered Japan’s flat rate of 30% to be sufficiently high to deter impoverished people from seeking medical care. However, changing this co-payment system requires ample consideration [41]. The respondents were concerned about Japan’s acutely rising health-care expenditures and believed that the government’s tight budgets would not allow any reduction in the co-payment proportion [42]. To meet total health-care costs, the government faces very limited options: either increasing revenue by raising taxes, or increasing the proportion of health-care expenditure paid by insurance premiums or as co-payments [43]. Many physicians considered the zero co-payments for individuals receiving social welfare to be a cause of overutilization and a moral hazard and that the system might require some remediation. However, measures for overall cost containment should be undertaken simultaneously: Japan’s fee-for-service reimbursement system for outpatient care allows primary care doctors and patients to overutilize health care [44]. As some respondents mentioned in our survey, another option would be to change the rate of co-payment according to the type of disease and treatment from the currently employed flat rate. It appears necessary for the Japanese government to consider a policy that prevents inappropriate use of outpatient care while securing access for low-income patients by mitigating the financial burden to receive care.

This study has several limitations. First, the qualitative data were obtained as written texts, not from in-depth interviews; that could have limited the depth of the ideas expressed by the respondents. However, 365 physicians answered that question and some gave long responses; we consider that we had sufficiently rich data to understand the participants’ perspectives. Second, the relatively low response rate indicates that physicians who were especially aware of their patients’ economic issues were more likely to have responded. We did not know the characteristics of the non-responders to the survey, which may affect our interpretation of the results. However, age and gender distributions of our respondents were comparable to those of Japan’s national data of primary care physicians [45]. We purposefully chose survey areas that had higher proportions of socially disadvantaged individuals. The transferability of our results should be interpreted in light of the respondents’ backgrounds.

Despite these limitations, the results of this study highlight primary care physicians’ perspectives on the issue of co-payments as a possible deterrent for low-income patients seeking health care. Under the current policy, Japan’s increasing number of socially disadvantaged people may not have equitable access to health care. Widening health disparities may threaten Japanese longevity, of which the country has long been proud. The results of this study imply that the awareness of the possibly widening inequity in health-care should be raised both among primary care physicians and policy makers.

## Conclusions

Through their experience of having dealt with financially deprived patients, the majority of the primary care physicians surveyed agreed with lowering co-payments for such patients. However, the remaining participants disagreed with or were uncertain about such a move, and they believed its feasibility to be an area of huge concern. It may be necessary to raise awareness of patients’ socioeconomic status among primary care physicians as a possible deterrent to undergoing care. At the same time, to maintain health-care equity, policy makers should consider balancing co-payments among individuals with differing financial levels and health-care needs.
